# A postbiotic fermented oat gruel may have a beneficial effect on the colonic mucosal barrier in patients with irritable bowel syndrome

**DOI:** 10.3389/fnut.2022.1004084

**Published:** 2022-12-08

**Authors:** Olga Bednarska, Olga Biskou, Hans Israelsen, Martin E. Winberg, Susanna Walter, Åsa V. Keita

**Affiliations:** ^1^Department of Gastroenterology, Linköping University Hospital, Linköping, Sweden; ^2^Department of Biomedical and Clinical Sciences, Linköping University, Linköping, Sweden; ^3^Nordic Rebalance A/S, Hillerød, Denmark; ^4^Department of Health, Medicine, and Caring Sciences, Linköping University, Linköping, Sweden

**Keywords:** postbiotics, IBS–irritable bowel syndrome, intestinal barrier, fermented oats, gut permeability

## Abstract

**Background:**

Impaired intestinal permeability and microbial dysbiosis are important pathophysiological mechanisms underlying irritable bowel syndrome (IBS). ReFerm^®^, also called Profermin^®^, is a postbiotic product of oat gruel fermented with *Lactobacillus plantarum* 299v. In this study, we investigated whether ReFerm^®^ has a beneficial effect on the intestinal epithelial barrier function in patients with IBS.

**Materials and methods:**

Thirty patients with moderate to severe IBS-diarrhoea (IBS-D) or IBS-mixed (IBS-M) were treated with enema containing ReFerm^®^ or placebo. The patients underwent sigmoidoscopy with biopsies obtained from the distal colon at baseline and after 14 days of treatment with ReFerm^®^ or placebo twice daily. The biopsies were mounted in Ussing chambers, and paracellular and transcellular permeabilities were measured for 120 min. In addition, the effects of ReFerm^®^ or placebo on the epithelial barrier were investigated *in vitro* using Caco-2 cells.

**Results:**

ReFerm^®^ reduced paracellular permeability (*p* < 0.05) and increased transepithelial resistance (TER) over time (*p* < 0.01), whereas the placebo had no significant effect in patients. In ReFerm^®^-treated Caco-2 cells, paracellular and transcellular permeabilities were decreased compared to the control (*p* < 0.05) and placebo (*p* < 0.01). TER was increased in Caco-2 ReFerm^®^-treated cells, and normalised TER was increased in ReFerm^®^-treated Caco-2 cells compared to control (*p* < 0.05) and placebo-treated (*p* < 0.05) cells.

**Conclusion:**

ReFerm^®^ significantly reduced paracellular permeability and improved TER in colonic biopsies collected from patients with IBS and in a Caco-2 cell model. Our results offer new insights into the potential benefits of ReFerm^®^ in IBS management. Further studies are needed to identify the molecular mechanisms underlying the barrier-protective properties of ReFerm^®^.

**Clinical trial registration:**

[https://clinicaltrials.gov/], identifier [NCT05475314].

## Introduction

Irritable bowel syndrome (IBS), a chronic visceral pain disorder with a female predominance, is characterised by recurrent abdominal pain and disturbed bowel habits and is often accompanied by extraintestinal symptoms, such as anxiety and depression (1). The global IBS prevalence is approximately 4% according to Rome IV criteria (2), rendering IBS as one of the most common disorders of the gut-brain axis (3, 4). Additionally, IBS accounts for a substantial economic and individual burden (5–8). Patients with IBS are categorised into subgroups based on the predominant stool form: IBS with predominant constipation (IBS-C), IBS with predominant diarrhoea (IBS-D), IBS with mixed bowel habits (IBS-M), and unclassified IBS (IBS-U) (3), Progression from one subgroup to another may occur over time (9, 10). Owing to unclear pathophysiology and heterogeneity, IBS still lacks effective treatment. Nevertheless, increasing evidence strongly supports that disturbed intestinal barrier function and altered microbiota and immune activation contribute to IBS pathophysiology (11, 12). The intestinal barrier dysfunction and increased mucosal permeability have been mostly implicated in IBS-D, as well as in post-infectious IBS (PI-IBS) (13). And were associated with alterations in tight junction proteins (14). The national and international guidelines for IBS treatment include dietary advice and oral intake of probiotics as a first-line treatment; however, no specific probiotic species have been identified yet (15).

Probiotics, mainly *Lactobacilliace* and *Bifidobacteria*, have been shown to improve the intestinal barrier function and regulate the increased intestinal permeability by restoring the mucosal layer (13, 16–19), and inhibit pathogen invasion of and adhesion to epithelial cells through niche competition and production of numerous metabolites (20, 21). *Lactobacilli* strains have been shown to improve stool consistency and intestinal transit in murine IBS models (22, 23). *Lactobacillus L. plantarum* 299v is one of the well-studied *L. plantarum* strains (24), with several specific characteristics, such as the ability to survive the passage through the gastrointestinal tract (25), the ability to attach to the colonic mucosa *via* mannose adhesion (26), and the ability to improve transepithelial resistance (TER) by increasing the production of tight junction proteins in a Caco-2 cell layer (27, 28). Furthermore, *L. plantarum* 299v enhances the production of short-chain fatty acids (SCFA), particularly acetic and propionic acids (29), and has antimicrobial properties against potential gastrointestinal pathogens (29), an immunomodulatory activity (23), and a mucoprotective activity by increasing mucus production from goblet cells (19). Owing to these properties, *L. plantarum* 299v has gained increasing attention in IBS research. Several studies have demonstrated a significant effect of *L. plantarum* 299v in alleviating global IBS symptoms, including abdominal pain and flatulence (30–32). However, the reported data are inconsistent, showing no significant differences in IBS symptoms, quality of life, or rectal sensitivity between *L. plantarum* 299v and placebo treatments (33, 34). A previous study has reported worsening of gastrointestinal symptoms in patients with IBS treated with the *L. plantarum* strain MF1298 compared to the placebo group (35).

Postbiotics are defined as the preparation of inanimate microorganisms and/or their components that confers a health benefit on the host on the gut environment (36). An increasing number of studies, mainly on *Lactobacilli* strains, have reported beneficial effects of postbiotics on human health. Compare *et al.* (37) have reported anti-inflammatory effects of *Lactobacillus casei* and its postbiotic mediators in *ex vivo* biopsy culture of post-infectious IBS-D. Notably, the effect of *Lactobacilli*-derived end-products was more robust than that of the probiotic strain. Microbial fermentation, a well-known food preservation method, produces lactic acid and a variety of cellular structures and metabolites, such as cell surface components, SCFAs, and bioactive peptides, which have been linked to human health (36). Tarrerias *et al.* (38) demonstrated in an uncontrolled study that fermented postbiotic products of the *Lactobacillus acidophilus* (strain LB) improved both IBS symptoms, such as abdominal pain, bloating, stool number and consistency, as well as the quality of life of patients with IBS-D.

ReFerm^®^ (also called Profermin^®^) is a food product of oat gruel fermented with *L. plantarum* 299v, containing high amounts of SCFA and other microbial metabolites. A randomised controlled study has shown that ReFerm^®^ significantly reduces symptoms in patients with mild-to-moderate ulcerative colitis (39). Therefore, the present study aimed to evaluate the effect of ReFerm^®^ on intestinal barrier function in patients with IBS. In addition, we investigated the direct effect of ReFerm^®^ on TER and paracellular and transcellular permeabilities *in vitro* using the Caco-2 model.

## Materials and methods

### Study design

**FIGURE 1:**
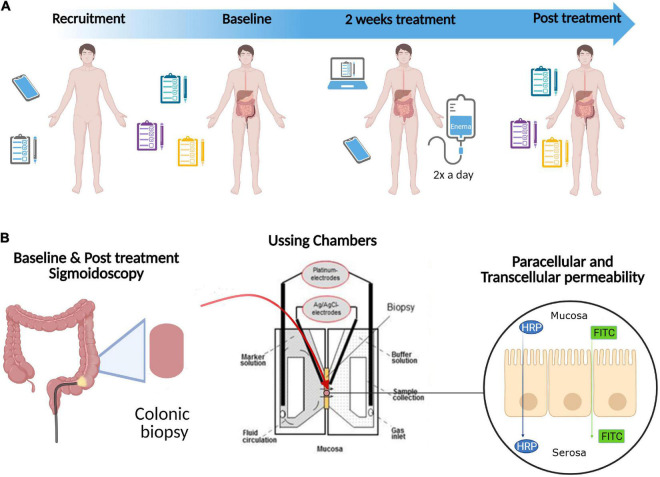
Study design. **(A)** Potential participants were screened based on the inclusion and exclusion criteria (Table 1) during telephone interviews (*Recruitment*). Eligible patients were then randomly allocated to one of two study arms: Referm^®^ or placebo. The patients completed symptom questionnaires and underwent sigmoidoscopy with biopsies obtained from the distal colon at *Baseline* and after 14 days of intervention (*2 weeks treatment*) with ReFerm^®^ or placebo enema twice a day (*Post treatment*). During the *2 weeks treatment*, the patients were given a check-up call by a principal investigator (OBe) twice per week and completed daily web questionnaire. **(B)** Biopsies obtained from the distal colon during *Baseline and Post treatment sigmoidoscopy* were mounted in Ussing chambers to determine paracellular [using fluorescein isothiocyanate (FITC)-dextran 4000] and transcellular [using horseradish peroxidase (HRP)] permeabilities of the intestinal mucosa. In addition, the direct effect of ReFerm^®^ and placebo on paracellular and transcellular permeabilities *in vitro* were examined using the Caco-2 model (not shown in this figure). Created with BioRender.com.

We conducted a single-blinded, randomised experimental study (Figure 1). Potential participants were screened based on the inclusion and exclusion criteria (Table 1) during telephone interviews. As the patients were their own controls, self-reported allergy was not an exclusion criterion as long as both interventions were carried out during unchanged allergic exposure.

**TABLE 1:** Inclusion and exclusion criteria for the study.

Inclusion criteria	Exclusion criteria
• Confirmed IBS-D or IBS-M according to Rome IV criteria • Moderate to severe IBS according to IBS-SSS score (≥175 p) • Age 18–70 years • Fluency in written and spoken Swedish	• Organic gastrointestinal disease • Previous major gastrointestinal operation (apart from appendectomy and cholecystectomy) • Psychiatric disease (bipolar disease, schizophrenia) • NSAID intake less than 2 weeks prior to endoscopy • Self-reported pregnancy

IBS-D, irritable bowel syndrome with predominant diarrhoea; IBS-M, IBS with mixed bowel habits; IBS-SSS, IBS severity scoring system; NSAID, non-steroidal anti-inflammatory drugs.

Patients were then randomly allocated to one of two study arms: Referm^®^ or thickened water (Thick-it^®^, commercially available; Kent Precision Foods Group, Inc., Muscatine, IA, USA) used as placebo. The patients underwent sigmoidoscopy with biopsies obtained from the distal colon at baseline and after 14 days of intervention with ReFerm^®^ or placebo enema twice a day. The enema was administered rectally, in the left side position, and retained for as long as possible (at least 10 min) both in the left-sided and supine body position to activate retrograde peristalsis. To assess clinical improvement of symptoms, questionnaires were completed twice: before and after the intervention. During the 14 days of the intervention, the patients completed daily questionnaires, as described in the questionnaire section. To improve compliance with the study intervention, the patients were given a check-up call by a principal investigator (OBe) twice per week during the intervention period.

### Patients

The study was approved by the Committee of Human Ethics, (Dnr 2020-03485), and all participants provided written informed consent. The study was registered at clinicaltrials.gov with the ID number NCT05475314.

Thirty patients (five men) meeting the Rome IV criteria (2), with a mean IBS duration of 13 years (range 2–40 years), were recruited from the Gastroenterology Department, University Hospital, Linköping between December 2020, and April 2021. The patients had a mean age of 37 years (range 19–55 years) and a mean body mass index (BMI) of 26 (range 18–41). The patients were classified according to predominant bowel habits into IBS-D (*n* = 8) and IBS-M (*n* = 22) according to the Rome IV criteria and had moderate-severe IBS with a mean symptom severity score of 332.5 (range 180–488) according to IBS-SSS (40). They were randomly allocated to ReFerm^®^ (18 patients, two men) or placebo (12 patients, three men) arms. There were no significant differences between the groups in terms of age, BMI, or disease severity. Four (no men) patients and two (one man) patients dropped out of the ReFerm^®^ and placebo arms, respectively.

### The intervention product ReFerm^®^

#### The clinical intervention product ReFerm^®^

ReFerm^®^ was manufactured as previously described (41). Following the fermentation process, the product is tested for pH and colony-forming units (CFU) of *Enterobacteriaceae*, yeasts/moulds, and *L. plantarum* 299v. The pH must be <4.0, the CFU of *Enterobacteriaceae* and yeasts/moulds must be <100/ml, and the CFU for *L. plantarum* 299v must be >10^8^ immediately after completion of fermentation. The energy content of 100 ml ReFerm^®^ is 58 kcal (240 kJ), from 1.6 g protein, 9.8 g carbohydrate, and 0.9 g fat. Each ReFerm^®^ package contained 250 ml.

#### The *in vitro* intervention product ReFerm^®^

For the *in vitro* experiments, 250 ml of ReFerm^®^ packages were heat-treated at 80°C, and the liquid ReFerm^®^ inside the package was kept at 80°C for 90 min, followed by cooling at 20°C. The CFU in heat-treated ReFerm^®^ was confirmed to be <1 per ml.

### The placebo product Thick-it^®^

To mimic the intervention product, which is characterised by higher viscosity than pure water, a product of thickened water (Thick-it^®^ mildly thick, Kent Precision Foods Group, Inc.; commercially available) was chosen as a placebo to mimic the viscosity. This product contains artesian mineral water and ≤2% xanthan gum, calcium chloride, malic acid, potassium benzoate, potassium sorbate (to preserve freshness), sodium hexametaphosphate, and disodium ethylenediaminetetraacetic acid (EDTA). The energy content of 237 ml Thick-it^®^ (one package) was 5 kcal (21 kJ) from 0 g protein, 1 g carbohydrate, and 0 g fat.

### Questionnaires

All questionnaires were completed twice, before and after the intervention, during the study visit for sigmoidoscopy. The web questionnaire was completed daily during the 14 days of intervention.

#### Irritable bowel syndrome severity scoring system

Irritable bowel syndrome-severity scoring system was used to assess IBS symptoms and distinguish between patients with severe IBS upon inclusion in the study. The scoring system incorporated five items: abdominal pain severity, pain frequency, bowel distension, bowel dysfunction, and quality of life/global wellbeing. The maximum total possible score was 500. Mild, moderate, and severe symptom burdens were indicated by score ranges of 75–175, 175–300, and >300, respectively. A reduction of 50 points in IBS-SSS is considered a significant improvement in treatment studies (40).

#### Gastrointestinal symptom rating scale-irritable bowel syndrome

Gastrointestinal symptom rating scale-IBS is a self-assessment instrument used to assess the symptoms of IBS (42). The questionnaire includes thirteen items, each assessed by a seven-point rating scale (1–7), grouped into five symptom clusters: abdominal pain (2 items), bloating (3 items), constipation (2 items), diarrhoea (4 items), and satiety (2 items). The data were analysed by cluster or as a total score for all 13 items.

#### Visceral sensitivity index

Visceral sensitivity index consists of a 15-item scale to measure gastrointestinal symptom-specific anxiety by assessing cognitive, affective, and behavioural responses to fear of gastrointestinal sensations, symptoms, and the context in which these occur (43).

#### Hospital anxiety and depression scale

Hospital anxiety and depression scale was used to estimate the states of depression and anxiety (44). The scale consists of seven items for depression (HADS-D) and anxiety subscales (HADS-A), with scores on each subscale ranging from 0 to 21. Cut-off values are indicated as ≥8 for subclinical (suspicious) anxiety or depression and ≥11 as definite cases on both the HADS-D and HADS-A, respectively (45).

#### Short health scale

Short health scale is a validated instrument for measuring subjective health in patients with IBS. The questionnaire included four items: symptom burden, daily functioning, disease-related worry, and general wellbeing. The answer to each item is given as a mark on a 0–100 mm visual analogue scale; hence, the maximum score for each item is 100, and the maximum score for all items is 400 (46).

#### Daily web questionnaire

To report daily changes in IBS symptom burden and emotional state, an electronic version of a short end-of-day questionnaire consisting of six questions about abdominal pain severity, frequency, consistency of bowel movements during the day, symptom burden, daily function, disease-related worry, and general wellbeing (in-house, non-validated).

### Sigmoidoscopy

Flexible sigmoidoscopy was performed after routine preparation with an enema for bowel emptying. Sigmoidoscopy was performed without sedation, with a scope inserted approximately 30–40 cm orally from the linea dentata. Colonic biopsies were obtained with biopsy forceps without a central lance and directly placed in ice-cold oxygenated Krebs buffer (115 mM NaCl, 1.25 mM CaCl_2_, 1.2 mM MgCl_2_, 2 mM KH_2_PO_4_, and 25 mM NaHCO_3_, pH 7.35). Sigmoidoscopy was performed twice for each participant at baseline and after 14 days of enema treatment with ReFerm^®^ or placebo.

### Ussing chamber experiments on colonic biopsies

Colonic biopsies were mounted on Ussing chambers (47) as previously described (48). After 30 min of equilibration, 250 μM of the paracellular probe fluorescein isothiocyanate (FITC)-dextran 4,000 (Sigma-Aldrich, St. Louis, MO, USA) and 10 μM of the 44 kD transcellular probe horseradish peroxidase (HRP) (type VI; Sigma-Aldrich) were added to the mucosal sides. Samples from the serosal side were collected at 0, 30, 60, 90, and 120 min. The transepithelial potential difference (PD), TER, and short-circuit current (Isc) across the tissues were monitored throughout the experiments to ensure tissue viability.

### *In vitro* experiments

Caco-2 cells were grown in high-glucose Dulbecco’s modified Eagle’s medium (DMEM, Gibco) supplemented with 10% v/v foetal bovine serum (FBS, Gibco), 1% L-glutamine (Gibco, Waltham, MA, USA), and 1,000 units/ml or μg/ml of penicillin/streptomycin (penicillin/streptomycin), respectively, at 37°C in a 5% CO_2_ incubator. Caco-2 cells were plated at a density of 2.5 × 10^4^ cells per Transwell on a 24-well plate (Corning Inc., Corning, NY, USA) in a complete growth medium (DMEM, 10% FBS, 1% L-glutamine, and penicillin/streptomycin). The plated cells were allowed to grow for 14 days at 37°C in a 5% CO_2_ incubator. The medium was replaced every two to 3 days, and at the same time, TER was measured using Millicell^®^ ERS-2 (MilliporeSigma, Burlington, MA, USA) combined with the MERSSTX01 electrode to assess cell growth.

On the day of the experiment, the cells and wells were washed with D-PBS (Gibco) to remove phenol red residues. High-glucose DMEM without phenol red (Gibco) was supplemented as previously described. A full growth medium without phenol red was added to both the apical and basal sides of Caco-2 cells. TER was measured to determine baseline. Once TER was measured, the media in the transwells were replaced with fresh media (control) and thickened water diluted (1:20) in full growth media (placebo). Owing to the high content of fibres in ReFerm^®^, it needed to be diluted. ReFerm^®^ was centrifuged at 300 × *g* for 5 min, and the supernatant was diluted 1:20 in full growth media and used as ReFerm^®^ treatment. All treatments contained 250 μM FITC-dextran-4000 and 10 μM HRP to allow measurement of paracellular and transcellular permeabilities. The media within the wells were collected and replaced at 1, 2, 4, 6, 20, and 24 h, and TER was measured at the same time.

### Determination of paracellular passage

To determine the fluorescence intensity of FITC-dextran in the collected samples (either from the Ussing chamber or cell culture), samples were plated on a black 96-well plate (49). The intensities of the samples and known standards were measured at 488 nm using a VICTOR*™* X3 multileader plate reader. The optical density of the known standards was used to create a standard curve that was used to determine the concentrations of unknown samples.

### Determination of transcellular passage

Horseradish peroxidase was measured using the QuantaBlu*™* Fluorogenic Peroxidase Substrate Kit (Pierce, Thermo Fisher Scientific, Waltham, MA, USA), as previously described (49) and passage was further determined using VICTOR*™* X3.

### Statistical analysis

Parametric data are presented as mean ± SD, and comparisons between groups were performed using Student’s *t*-test and analysis of variance (ANOVA) test. Non-parametric data are presented as median [25–75th interquartile range], and comparisons between groups were performed using the Kruskal–Wallis and Mann–Whitney *U*-tests. Wilcoxon matched-pair signed-rank tests were used for paired comparisons. Correlation testing was performed using Spearman’s correlation test. Statistical analysis was performed using GraphPad Prism 9 version 9.1.2. Statistical significance was set as *p* < 0.05.

## Results

### Questionnaires

Due to the limited number of participants in both investigation groups, the questionnaire data were mostly non-parametric according to the Shapiro–Wilk test; therefore, the Wilcoxon test was used to compare the questionnaire data before and after the intervention. Differences in questionnaire data before and after intervention are presented in Table 2. Five patients in the ReFerm^®^ group and four in the placebo group reported significant improvement after the intervention, defined as a reduction of at least 50 points in the IBS-SSS.

**TABLE 2:** Differences in questionnaire data before and after intervention in placebo and ReFerm^®^ groups.

	Placebo group	ReFerm^®^ group
IBS-SSS	*z* −2.60, *p* < 0.05	NS
GSRS-IBS	NS	NS
VSI	NS	*z* −2.69, *p* < 0.05
HADS	NS	NS
SHS	*z* −2.19, *p* < 0.05	*z* −2.73, *p* < 0.05
DWQ	NS	NS

Numbers given in *z* score (standard score, the number of standard deviations a given data point lies above or below mean) and *p*-value. IBS-SSS, IBS severity scoring system; GSRS-IBS, gastrointestinal symptom rating scale-IBS; VSI, visceral sensitivity index, HADS, hospital anxiety and depression scale; SHS, short health scale; DWQ, daily web questionnaire; NS, not significant.

### Electrophysiology

**FIGURE 2:**
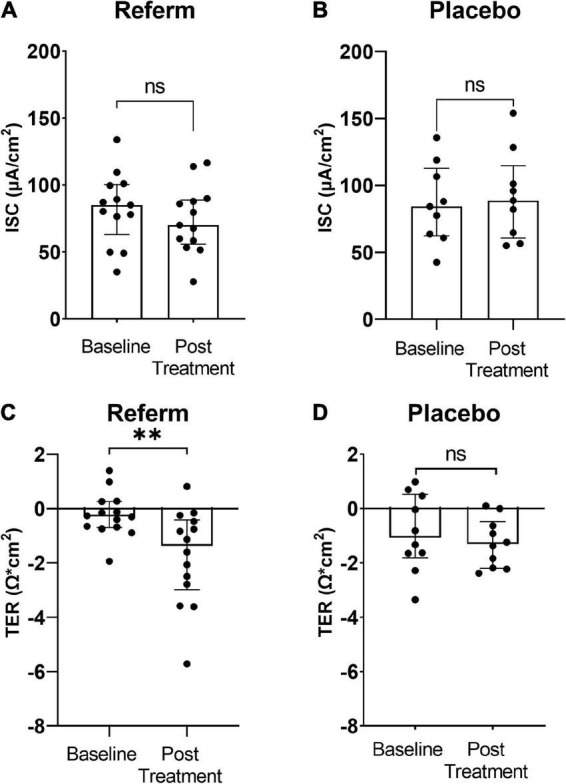
The effect of fermented oat gruel (ReFerm^®^) or placebo interventions on short-circuit current (Isc) and transepithelial resistance (TER) in irritable bowel syndrome (IBS). Patients with IBS were allocated to the ReFerm^®^ arm (*n* = 18) or the placebo arm (*n* = 12). Biopsies were collected at baseline and after the intervention. **(A)** Isc at baseline and after ReFerm^®^ treatment. **(B)** Isc at baseline and after placebo intervention not significant (ns). **(C)** TER, difference 30–90 min at baseline and after ReFerm^®^ treatment ^**^p < 0.01. **(D)** TER, difference 30–90 min at baseline and post placebo intervention. Data are presented as the median and interquartile range (IQR). *n*, number of patients.

Potential difference was stable in all biopsies mounted in Ussing chambers throughout the experiments (data not shown). Similarly, no significant differences were observed in Isc between the ReFerm^®^ (Figure 2A) and placebo groups (Figure 2B). The differences in TER over time (30–90 min) were significantly decreased in the ReFerm^®^ arm after the intervention compared with baseline (*p* < 0.01; Figure 2C); however, there was no significant change in TER over time in the placebo group (Figure 2D).

### Intestinal permeability

Paracellular and transcellular intestinal permeabilities of the biopsies mounted in Ussing chambers were measured in parallel with the *ex vivo* electrophysiological measurements.

#### ReFerm^®^ intervention reduces the paracellular permeability

**FIGURE 3:**
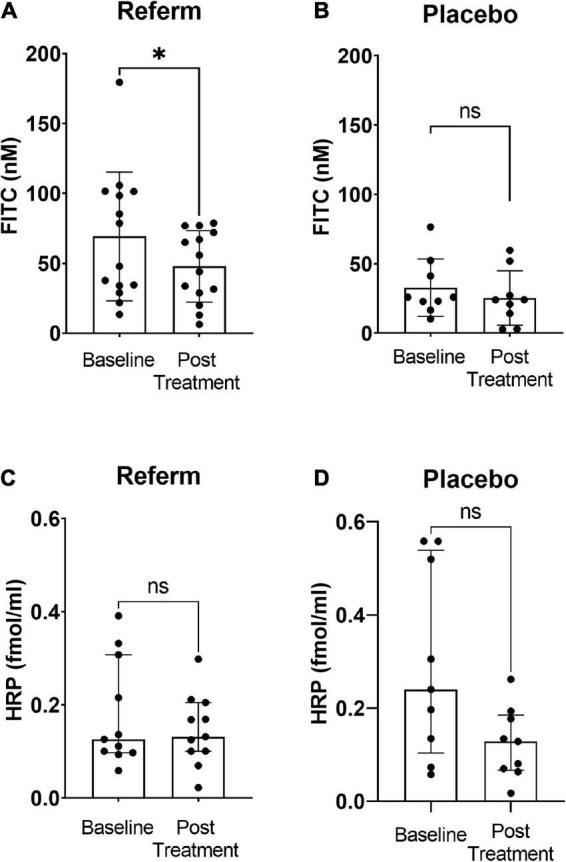
Effects of fermented oat gruel (ReFerm^®^) or placebo on the intestinal permeability of patients with irritable bowel syndrome (IBS). Patients with IBS were allocated to the ReFerm^®^ arm (*n* = 18) or the placebo arm (*n* = 12). Biopsies were collected at baseline and after the intervention. **(A)** Paracellular permeability to fluorescein isothiocyanate (FITC)-dextran 4,000 decreases significantly after ReFerm^®^ intervention *p < 0.05. **(B)** Placebo intervention does not alter the paracellular permeability not significant (ns). **(C)** ReFerm^®^ intervention does not affect the transcellular permeability to horseradish peroxidase (HRP). **(D)** Placebo intervention does not affect the transcellular permeability. Data are presented as the median and interquartile range (IQR). *n*, number of patients.

We observed a significant reduction in the paracellular permeability after treatment with ReFerm^®^ compared to that before the intervention (*p* < 0.05; Figure 3A). No significant difference was observed between baseline and placebo treatments in the placebo groups (Figure 3B).

#### ReFerm^®^ intervention does not affect the transcellular permeability

No significant differences were observed in the transcellular permeability between baseline and post-intervention in neither the ReFerm^®^ group (Figure 3C) nor the placebo group (Figure 3D).

### Effects of ReFerm^®^ on permeability in Caco-2 cells

#### ReFerm^®^ increases transepithelial resistance in Caco-2 cells

**FIGURE 4:**
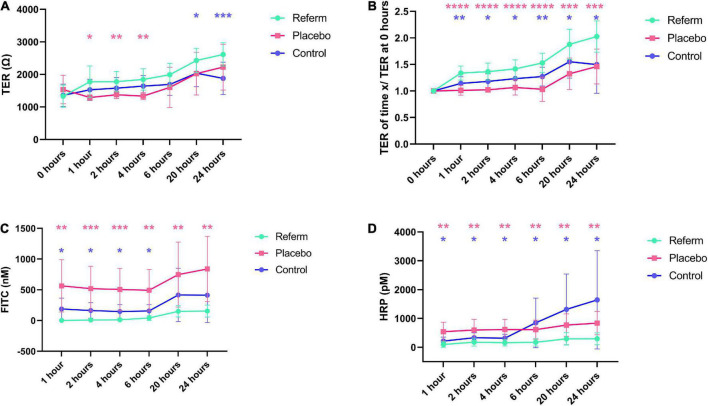
Effects of the fermented oat gruel, ReFerm^®^, on the barrier function over time *in vitro*. **(A)** Transepithelial resistance (TER) in Caco-2 cells treated with ReFerm^®^, placebo, or untreated control. **(B)** TER normalised against the baseline measurements (0 h). **(C)** The paracellular permeability is decreased in ReFerm^®^-treated cells compared to untreated or placebo-treated cells. **(D)** The transcellular permeability is significantly decreased in ReFerm^®^-treated cells compared to untreated controls. Data are presented as mean ± standard deviation (SD) (*n* = 12 wells per condition per time point) *p < 0.05, **p < 0.01, ***p < 0.001, ****p < 0.0001. Pink stars represent the comparison between ReFerm^®^ and placebo, while the blue stars represent the comparisons between in ReFerm^®^- and control.

In all treatments, we observed a similar pattern of the increase in TER over time (Figure 4A). We observed a significant increase in TER in Caco-2 cells treated with ReFerm^®^ (*p* < 0.0001), whereas placebo-treated cells and controls only showed significant increases at specific time points, compared to their respective baselines (Figure 4A).

To compensate for the variability between the experiments, the results were normalised to the baseline measurements for each Transwell. The normalised TER showed an increasing pattern in all treatments, similar to TER. Compared to baseline, a significant increase (*p* < 0.0001) over time was observed in ReFerm^®^-treated cells (Figure 4B). Placebo-treated cells and control cells only showed a significant increase at specific time points (Figure 4B). Normalised TER was significantly increased in ReFerm^®^-treated Caco-2 cells compared to control cells (*p* < 0.05, *p* < 0.01, depending on the time point; Figure 4B) and placebo-treated cells (*p* < 0.001 or *p* < 0.0001; Figure 4B).

Collectively, these results indicated that ReFerm^®^ resulted in increased TER over time compared to the baseline, as well as increased normalised TER compared to untreated or placebo-treated Caco-2 cells.

#### ReFerm^®^ decreases the paracellular permeability in Caco-2 cells

We observed that the paracellular permeability increased over time in all treatment groups (Figure 4C). There was a significant increase in paracellular permeability at 6 (*p* < 0.05), 20 (*p* < 0.01), and 24 h (*p* < 0.01) compared to that at 1 h in ReFerm^®^-treated cells. The paracellular permeability of ReFerm^®^-treated cells was significantly lower than that of the control cells for the first 6 h (*p* < 0.05) and lower at 20 and 24 h. Similarly, the paracellular permeability of ReFerm^®^-treated cells was significantly lower (*p* < 0.01 or *p* < 0.001) than that of the placebo-treated cells (Figure 4C). These results indicate that ReFerm^®^ treatment resulted in decreased paracellular permeability compared to placebo treatment or no treatment (control cells).

#### ReFerm^®^ reduces the transcellular permeability in Caco-2 cells

We observed that the transcellular permeability increased over time in all treatment groups (Figure 4D). However, the transcellular permeability of ReFerm^®^-treated cells was significantly lower overtime than that of the control cells (*p* < 0.05; Figure 4D). Similarly, the transcellular permeability of ReFerm^®^-treated cells was significantly lower (*p* < 0.01) than that of the placebo-treated cells overtime. Therefore, ReFerm^®^ treatment resulted in decreased transcellular permeability compared to placebo treatment or no treatment.

### Relation between gut findings and clinical data

There was no correlation between age, BMI, or disease duration of patients with IBS and any of the parameters investigated in the study (results not shown). No significant correlation was observed between the questionnaire results and mucosal barrier function parameters investigated in this study (results not shown).

## Discussion

In the present study, we demonstrated that the postbiotic food product of oat gruel fermented with *L. plantarum* 299v diminished the paracellular permeability in colonic biopsies of patients with IBS. We confirmed the results *in vitro* and showed that ReFerm^®^ decreased the paracellular and transcellular permeabilities and increased TER in Caco-2 cells.

Over the last few decades, there has been remarkable progress in the understanding of the pathophysiology of IBS, and recent studies have focussed on the interplay between central and peripheral mechanisms along the gut-brain axis with no single mechanism being independent or prevalent. There is mounting evidence of a disturbed intestinal barrier function in IBS (13, 50–52) as well as a correlation between increased intestinal permeability and IBS symptom severity (15). Various attempts have been made to regulate intestinal permeability to reduce IBS symptom burden (30–32, 38, 53–57), with disparate results (33–35, 50), reflecting the multifaceted pathophysiology of IBS.

The relationship between lower biodiversity and microbiota dysbiosis (decrease in probiotic species and abundance of pathogenic species) (58, 59) and altered bowel function has been demonstrated in IBS (24, 55, 60–62). The favourable effect of probiotics on IBS symptoms is considered to be strain specific (56, 57). *L. plantarum*, a well-documented probiotic species that has been investigated for more than 30 years (24), has beneficial effects in IBS, inflammatory bowel disease, gastrointestinal infections, iron deficiency anaemia, and depression (24). In a recent meta-analysis, a subgroup analysis of randomised clinical trials suggested a prominent effect of the *L. plantarum* strain 299v in alleviating global IBS symptoms (56). However, a more recent systematic review and network meta-analysis has suggested a prominent efficacy of *Bifidobacterium coagulans* in diminishing abdominal pain in IBS, while *L. plantarum* ranked first in improving the quality of life of IBS patients (55).

Postbiotics were previously defined as the metabolites obtained during fermentation processes and have been shown to confer beneficial effects on the gut environment (36–38). At present, probiotics are defined as the entire composition of inanimate microbial cells and metabolites (36). The postbiotic ReFerm^®^ contains both the *Lactobacilli* and the excreted metabolites during the fermentation in the production process. ReFerm^®^ has been shown to effectively reduce the symptoms of ulcerative colitis in clinical studies (39, 41). In a 24-week uncontrolled intervention study in patients with active ulcerative colitis, ReFerm^®^ improved the score of the simple clinical colitis activity index (SCCAI), which covers symptoms such as bowel frequency, blood in faeces, faeces consistency, urgency of defaecation, and general wellbeing (41). Additionally, ReFerm^®^ exhibited a high safety profile (41). A randomised controlled trial on active ulcerative colitis has shown that ReFerm^®^ reduces SCCAI scores at a statistically and clinically significant level in patients with mild-to-moderate ulcerative colitis with a flare-up (39). Preliminary data from patients with IBS showed no difference in responses between ReFerm^®^ with heat-inactivated *L. plantarum* 299v and ReFerm^®^ with live *L. plantarum* 299v. Therefore, we assumed that this beneficial effect of ReFerm^®^ on the intestinal barrier was probably due to the presence of microbial metabolites, such as high amounts of SCFA, which are known to improve the integrity of the intestinal mucosa (62). However, in our study, the observed beneficial effect of ReFerm^®^ on the intestinal barrier in colonic biopsies collected from patients with IBS, which was also confirmed in *in vitro* experiments, was not substantiated by clinical data. We could not demonstrate either significant clinical improvement after ReFerm^®^ intervention using questionnaire data or the relationship between the gut findings and clinical data. We hypothesised that the rectal administration of ReFerm^®^ implemented in our study may have contributed to the aggravation of IBS symptoms, such as loose stools or straining. Nevertheless, the study application route was intentional, aiming to ensure the accuracy of the subsequent Ussing chamber experiments.

Our study has several strengths; this was a prospective, single-blinded study where the intervention product and placebo were administered in a controlled manner, and systematically audited by a principal investigator (OBe) during telephone check-ups. The products were applied rectally to warrant direct exertion of their effects on the examined mucosa. The *ex vivo* results were subsequently reproduced in a controlled laboratory environment using *in vitro* experiments. Moreover, because there was an effect on the transcellular permeability *in vitro*, an effect on HRP passage in the *ex vivo* situation was expected; however, the transcellular permeability remained unchanged. This may be because the *in vitro* situation is a simplified version of the human epithelial barrier, which consists of several layers and many different cell types (63). In addition, the cell culture conditions do not mimic the intestinal environment. In the *in vitro* model, ReFerm^®^ was added directly onto the Caco-2 cells, followed by measurement of HRP passage at defined time points, while in the *ex vivo* situation, ReFerm^®^ was administered as an enema to the patients, and HRP passage was measured after mounting the biopsies in Ussing chambers. These methodological differences could also explain the discrepancy in the results. Nevertheless, our results provide important insights into the direct effects of ReFerm^®^ on epithelial barrier function. At present, there is broad evidence of the protective effect of *L. plantarum* species on junctional complexes of the intestinal barrier (64–67). However, future studies are needed to investigate the molecular mechanism of action of ReFerm^®^ both *in vitro* and *in vivo*.

Our study is a proof-of-concept study aimed at evaluating the mechanism of action of the study product and was not designed to measure its clinical effect. *Ex vivo* studies on Ussing chambers routinely require a smaller sample size for practical reasons, in contrast to clinical questionnaire studies. In our study, the intervention interval was considerably short due to the experimental application route, thus, prolonging the treatment duration in patients with IBS may have positively influenced the permeability results in our study. According to different guidelines, recommended interval for treatment with probiotics varies between 4–12 weeks (56). Gender disproportion is undoubtedly further limitations of the generalisability of our study. Sex and gender differences both in IBS prevalence, subtype, and severity are well documented as well as inequality in health-seeking behaviours between men and women (68). This might explain the obstacles we faced while attempting to obtain equal gender representation in our study. Therefore, further studies with proper, oral administration of the intervention product in a larger, representative, gender-balanced IBS population and preferably longer treatment courses are needed to evaluate the clinical effect of the product in patients with IBS.

A putative limitation of this study might be the choice of placebo substance. To warrant the blinding of the patients to the study product, we aimed to find a sham product neutral to the colonic milieu but still different from plain water, as the patients were informed about the study product being based on oats. We selected thickened water to diminish the number of supplements with potential effects on the colonic mucosa. Xanthan gum, present at low doses in placebo products, is a polysaccharide containing glucose, mannose, potassium glucuronate, acetate, and pyruvate residues. It is widely used in drug suspensions in aqueous media because of its ability to form a hydrophilic colloid and its resistance to digestive enzymes in the stomach and small intestine. In the colon, it is dissolved into oligo- and monosaccharides, used thereafter as an energy source by bacteria or degraded by colonic enzymes (69). It also has a protective effect against oxidative stress in Caco-2 cells (69). However, our placebo had a similar consistency but different colour and smell compared to ReFerm^®^, which might have influenced the blinding negatively. The plausible impact of our placebo’s compounds on the colonic environment may also be a cause for concern since the hydrophilic colloid could act as a mechanical barrier, thus affecting mucosal permeability. Furthermore, the placebo effect is remarkably large in IBS clinical trials (70). Although regular patient-doctor interactions during our study could give rise to a substantial placebo effect, no significant clinical improvement in IBS symptoms after any intervention was observed in our study.

In conclusion, this proof-of-concept study demonstrated the potential mucosal barrier-protecting properties of ReFerm^®^ on the colonic mucosa of patients with IBS *ex vivo* and *in vitro*. Despite the limitations discussed above, the study results are promising and offer new insights into the potential benefits of ReFerm^®^ in IBS management. Further studies are needed to identify the molecular mechanisms underlying ReFerm^®^ action and to investigate the presumed beneficial clinical effects of ReFerm^®^ on IBS symptoms.

## Data availability statement

The original contributions presented in this study are included in the article/supplementary material, further inquiries can be directed to the corresponding author.

## Ethics statement

The studies involving human participants were reviewed and approved by the Etikprövningsmyndigheten; Box: 2110 750 02; Uppsala. Contact: registrator@etikprovning.se. Telephone: 0046 10-475 08 00. The patients/participants provided their written informed consent to participate in this study.

## Author contributions

OBe, OBi, HI, SW, and ÅK conceived the study. MW developed the methodology. OBe and SW collected the patient sample material and clinical information. OBi, MW, and ÅK performed the experimental data collection. OBe collected the clinical data. OBe, OBi, and ÅK performed the data analysis and drafted the manuscript. HI, SW, MW, and ÅK performed the manuscript revision. All authors reviewed and approved the final version of the article.
